# Resilience in Women who Experience Domestic Violence

**DOI:** 10.1007/s11126-017-9529-4

**Published:** 2017-08-12

**Authors:** Konstantinos Tsirigotis, Joanna Łuczak

**Affiliations:** Department of Psychology, The Jan Kochanowski University in Kielce, Piotrków Trybunalski Branch, Słowackiego 114/118 str, 97-300 Piotrków Trybunalski, Poland

**Keywords:** Resilience, Domestic violence, Women

## Abstract

Violence in the family constitutes a serious social and psychological problem with harmful consequences leading, among others, to changes in the psychological functioning of the victim and, secondarily, also the perpetrator. The aim of this study was to examine resilience in women experiencing domestic violence. The “Ego Resiliency Scale” (ERS) was used to study the group of women suffering domestic violence. The study group included 52 women aged 30–65 years (mean age: 40.15) using assistance of the Crisis Intervention Centre due to experienced domestic violence. They most often reported suffering psychological and physical violence, with the husband or intimate partner being the most common perpetrator. Study women experiencing domestic violence obtained significantly lower scores on the ERS. The lowest scores on the ERS were achieved by women suffering paternal violence, while the highest – by women experiencing violence on the part of the intimate partner. Resilience of study women suffering domestic violence was lower than resilience of the general population, i.e. individuals not experiencing domestic violence. Suffered violence inflicted by the father exerted the greatest adverse impact on resilience. It seems advisable to consider resilience in the process of providing women experiencing domestic violence with psychosocial help.

MOTTO: *… growth and pain are not mutually exclusive but rather inextricably linked in recovery from trauma and loss* (Saakvitne K.W., Tennen H., Affleck G.).

## Introduction

The phenomenon of violence in the family (domestic violence, DV and intimate partner violence, IPV) is a common one more often affecting women: violence against women is a global public health problem that besets approximately one third of women globally [cf. 1–3]. Violence in the family constitutes a serious social and psychological problem with harmful consequences for individuals who both experience violence and resort to it, leading, among others, to changes in the psychological functioning of the victim and, secondarily, also the perpetrator. Violence in the family may arise from emotionality disorders, personality disorders or psychotic disorders of the perpetrator, but it, certainly, also results from disturbances in relations between partners (regardless of the source of those disturbances) [4].

Although the notions of domestic violence and intimate partner violence are very similar to each other, as both assume experiencing violence inflicted by very close people, this study is going to deal with domestic violence as a broader concept, encompassing not only violence perpetrated by the intimate partner, but also by other family members. Violence in the family may concern all its members; it can also be of the mutual nature. In the case of physical violence, however, perpetrators tend to be men [5]. The essence of domestic violence is the use of an advantage of power or authority in order to harm the other family members. Browne and Herbert distinguish among physical, psychological and sexual violence, drawing attention to its active or passive forms and intensity. Domestic violence victims experience anxiety, suffering, helplessness, dispiritedness, and despair. Their bodies and psyches suffer acute injuries and are subject to processes of damaging and protracted stress and threat [4, 6].

Various authors understand domestic violence in a similar way. Domestic violence is defined as male aggression toward a female partner [7]. Domestic violence against women can be defined as any act or omission which, based on gender, causes death, physical, sexual or psychological injury, and moral damage to women; it can be inflicted by individuals with or without family ties who are either related by natural bonds, by affinity or by express will, including sporadic relationships [8]. Due to the significance of the problem, it also gained the attention of international organisations. Domestic violence is understood as all acts of physical, sexual, psychological or economic violence that occur within the family or domestic unit or between former or current spouses or partners, whether or not the perpetrator shares or has shared the same residence with the victim [9]. Authors of that point of view draw attention to the fact that it is not the current place of residence of the perpetrator or the ongoing nature of the relationship with the victim that are the most important. The United Nations Declaration on the Elimination of Violence Against Women [10] defines violence against women taking place in the family in the following way: “Physical, sexual and psychological violence occurring in the family, including battering, sexual abuse of female children in the household, dowry-related violence, marital rape, female genital mutilation and other traditional practices harmful to women, non-spousal violence and violence related to exploitation”. A similar phenomenon/term is the battering relationship defined as the repeated use of physical, sexual or verbal force by someone against his intimate partner [11].

In Poland, the definition contained in the national programme of violence prevention in the family is most commonly accepted. It describes violence as any action, intended and using an advantage of power, directed against a family member, which infringes his or her personal rights and interests, causing suffering and harm [12]. Another definition mentioned in Polish literature presents domestic violence as actions or glaring neglects on the part of a family member against the others, using an existing advantage of power or authority, or such an advantage created by circumstances, and causing harm or suffering to the victims, infringing their personal rights and, in particular, damaging their lives or (physical or mental) health [6].

Violence against women is not harmless; it produces numerous undesirable and adverse outcomes. Abused women are at increased risk of depression, anxiety, posttraumatic stress disorder and suicide, as well as physical problems [13]. Physical violence is associated with injuries (e.g. bruises, knife wounds, broken bones, headaches, back or pelvic pain, and death). Psychological abuse typically accompanies physical one, and the consequences of such abuse include depression, anxiety, posttraumatic stress disorder, and attachment disorders. Domestic violence has also been linked to increased adverse psychological and behavioural outcomes, such as smoking, drinking, taking drugs (i.e. substance abuse) or having unprotected sex, and other negative mental and physical health consequences [14].

For some time now, resilience or resiliency have more and more commonly been mentioned as yet another psychological resource. Research on resilience was initially conducted on children’s populations as first observations in that scope concerned children raised in families where at least one parent suffered from schizophrenia [cf. 15]; hence, world literature offers a considerable number of publications on resilience as a trait and/or process mainly in children. That was possibly due to the fact that children are characterised by higher flexibility and plasticity of the (central) nervous system and psychological functioning. It was only later that resilience began to be studied in adults. Many authors use the term “resiliency” as synonymous with the term “resilience” [cf. 16–18].

What the notions of “mental immunity”, “mental hygiene”, “mental resilience”, “emotional resilience” or “mental health resilience” have in common is the aim of broadening mental health research concepts, beyond risk factors for pathology, to include wellness enhancement and health promoting factors; a range of studies have suggested that “resilience” can be seen as synonymous with reduced “vulnerability”, with ability to adapt to adversity or to “cope” [19].

Resilience has been defined as the maintenance of healthy/successful functioning or adaptation within the context of a significant adversity or threat and is better characterised as a dynamic process, since individuals can be resilient to specific environmental hazards or resilient at one time but not another [7, 15]. It is a relative capacity for healthy adaptation to life adversities [20]. Resilience is considered a positive personality characteristic enhancing individual adaptation [21]. Resilience is conceived as a dynamic developmental process encompassing the attainment of positive adaptation within the context of significant adversity [22–25]. There are two critical conditions that are implicit within this conceptualisation of resilience: a) exposure to a significant threat, severe adversity or trauma; b) the achievement of positive adaptation despite major assaults on the developmental process [22, 24, 25]. Resilience is not something an individual “has” – it is a multiply determined developmental process, which is not fixed or immutable [25]. Resilience is also understood as a dynamic process in which psychological, social, environmental and biological factors interact to enable an individual at any stage of life to develop, maintain, or regain her/his mental health despite exposure to adversity [26].

The concept of resilience is used to describe the ability of the individual or group to face adversity positively, even when the environment is unfavourable. Resilience is characterised by human capacity to respond to everyday life demands in a positive way, despite adversities faced throughout the development of her/his life cycle, resulting in the combination of individual attributes and her/his family, social and cultural environments. Resilience is composed of two dimensions: resistance to destruction that relates to the ability to protect one’s integrity under strong pressure; and the ability to build or create a life worth living despite adverse circumstances [8].

Some researchers have investigated resilience as an individual trait or an epiphenomenon of adaptive temperament, while others as a process or force that drives a person to grow through adversity and disruption [19].

Resilience, previously described as the ability to achieve positive adjustment in spite of adversity, has more recently been defined as a process dependent on a range of ecological factors like family, school, peers, community responsibility, and social justice. Taking into account the multidimensional nature of resilience, some authors categorise resilience on different levels, which they refer to as “resilient”, “near-resilient”, and “non-resilient” [27].

Resilience may also be understood as the possibility of recovery from and overcoming of adversity, with the objective of strengthening and recovering of the individual, and making her/him emotionally stronger; the process of internal mobilisation, which triggers a movement of breaking away and of openness regarding the other with the aim of helping oneself and being helped, of overcoming the experience and finding new meaning for existence, is termed resilience [28].

To sum up, it can be stated that resilience is a set of personality characteristics, as well as skills and competences, which contribute to coping with stress, trauma, problems and adversities in life. It may be understood not only as a personality trait, but also as complex processes conditioning the adaptation and development of individuals and families facing various threats and adversities [29].

As arises from the above, the notion is consistent with the salutogenic approach in psychology. Resilience can be comprehended as one of the psychological resources enabling the human to cope with an (even extremely) difficult present situation and/or stress, as well as upon its occurrence.

As an interesting side note, it can be added that, although the coining of the term “resilience” is usually ascribed to Garmezy [15] and Werner [30], it was French neuropsychiatrist Boris Cyrulnik who dedicated his life to studies on the development of that concept [28].

Recently, in the framework of exploring psychological resources of women experiencing domestic violence, their emotional intelligence has been studied [4].

As a result of one of the scarce studies on the resilience of women suffering domestic violence, resilience was found to be high in women who had ended a battering relationship [11]. Another study indicated that women were surprised by an act of extreme violence, in which the aggressor tried to kill them, assault them and/or kill their children. The confrontation, which is the first step in the process of resilience, began once they faced the possibility of death: the resilience process started when the aggressor physically attempted to kill them, hurt and/or kill their children [8].

As it stems from the above, world literature offers a very small number of studies into resilience of women experiencing domestic violence.

The aim of this study has been to examine resilience in women suffering domestic violence.

## Methods

### Participants

The study group included 52 women aged 30–65 years (mean age: 40.15) using assistance of the Crisis Intervention Centre (CIC) due to experienced domestic violence. Women reported to the CIC on their own initiative or were referred there by an interdisciplinary team for the prevention of domestic violence and all had a “Blue Card”.^1^ The research was conducted by specialists (psychologists) at the start of the intervention, upon informing women about the aim of the study and obtaining their consent to participating in the research.

### Materials

In order to assess resilience, this study applied the Polish version of the “Ego Resiliency Scale (ER89)” (ERS) by Block and Kremen [17] as adapted by Kaczmarek [31]. The scale is composed of 14 items, on which the subject may take a position by choosing one of the four possible answers (Likert type scale). Both the original and Polish versions are characterised by high reliability and validity [17, 31].

### Statistical Analysis

The statistical analysis of received scores applied descriptive methods and statistical inference methods. In order to determine mean values for quantitative traits, arithmetic means (M) were calculated, while the standard deviation (SD) was assumed to be the dispersion measure. The statistical processing of received scores employed difference testing, multiple regression analysis, analysis of variance by ranks by Kruskal-Wallis (Kruskal-Wallis ANOVA by ranks), and multiple comparisons. For all the analyses, the maximum acceptable type I error was assumed at α = 0.05. Asymptotic two-sided test probability p was calculated and *p* ≤ 0.05 was considered statistically significant. The statistical analyses were performed by means of the *Statistica PL 13.0* statistical package [32].

## Results

Table 1 shows socio-demographic data and Table 2 – domestic violence data of the study group. The data indicate that most women were married (50%) and had higher education (40.38%). The study women reported that they had most often experienced psychological (96.15%) and physical (80.77%) violence, with the husband (73.08%) or intimate partner (17.31%) being the most common perpetrator. They had experienced violence for 2 to 25 years (mean time: 7.82). The study women suffering domestic violence obtained ERS scores (M: 34.615; SD: 6.250) significantly lower (*p* < 0.0000) than the general population not experiencing domestic violence (M: 39.564; SD: 5.711).

**Table 1:** Socio-demographic characteristics of the study group

Variable		n	%
Age	M± SD	39.46 ± 8.91	
	Range	21–65	
Marital status	Single	8	15.38
	Married	26	50.00
	Divorced	15	28.85
	Non-formalised relationship	3	5.77
Education	Primary	7	13.46
	Vocational	12	23.08
	Secondary	12	23.08
	Higher	21	40.38

**Table 2:** Violence data of the study group

Variable		n	%
Time	M± SD	7.82 ± 5.19	
	Range	2–25	
Types of Violence/Abuse^a^	Physical	42	80.77
	Psychological	50	96.15
	Sexual	18	34.62
	Economical	11	21.15
Perpetrator	Husband	38	73.08
	Intimate Partner	9	17.31
	Father	3	5.77
	Mother	2	3.84

^a^The sum of percentages may exceed 100% because the participants could report more than one type of violence/abuse

**Fig. 1:**
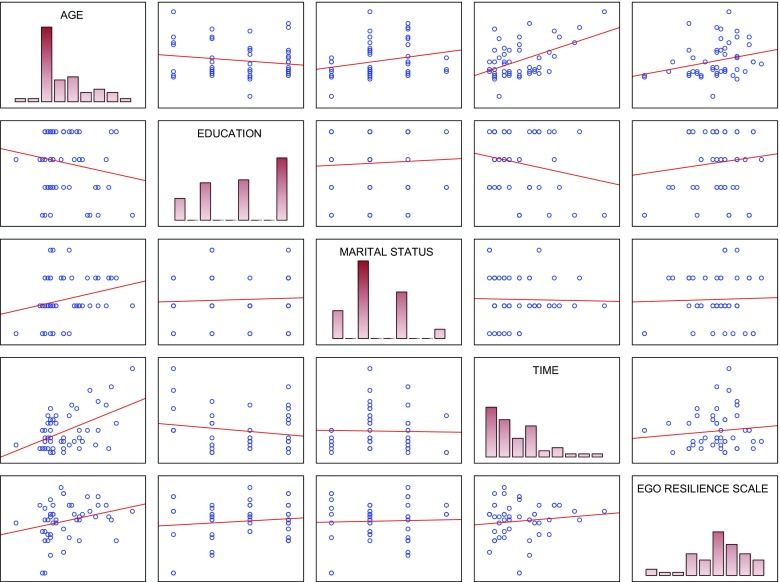
Scatterplot matrix of the studied variables

**Fig. 2:**
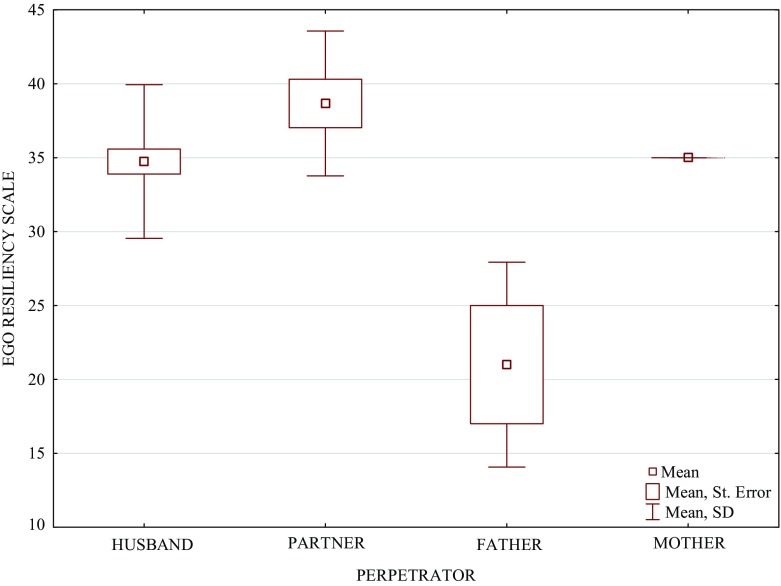
Resilience of study women regarding the perpetrator

In order to identify variables being significant predictors of resilience, stepwise multiple regression was used. Table 3 (and Fig. 1) present results of the stepwise multiple regression analysis, in which the dependent variable was resilience (ERS) and independent variables were socio-demographic and violence variables. All the variables were included in the initial regression equation. As shown in the table, all the variables remained in the regression equation, but not all of them turned out to be significant for resilience. Significant predictors were found to be age (β: 0.364; p: 0.002) and perpetrator (β: −0.247; 0.01). Therefore, the Kruskal-Wallis ANOVA by ranks was used (Table 4) to identify the perpetrator whose violence exerted the greatest impact on resilience intensity, while multiple comparisons (Table 5, Fig. 2) were performed to investigate possible differences in resilience intensity dependent on who committed violence against the women. As a result of the analyses, it was found that the lowest scores on the ERS were obtained by women suffering violence on the part of the father, while the highest – by women experiencing violence inflicted by the intimate partner. ERS scores of women experiencing violence on the part of the father, husband and intimate partner differed statistically significantly from one another.

**Table 3:** Stepwise multiple regression (Perpetrator-Resilience)

Dependent (Criterion) Variable: Resilience (ERS) Coefficient of Multiple Regression *R* = 0.399 Coefficient of Determination (R Square) R^2^ = 0.159 Corrected Determination coefficient (Adjusted R Square) R^2^ = 0.107 Significance of the regression eq. F(6, 45) = 3.057 *p* < 0.008 Std. error of the estimate: 5.877						
Independent (predictor) variables	Beta (β)	Std. Err. of Beta(β)	Std. B (β)	Std. Err. of B (β)	t (46)	p-level
Age	0.364	0.119	0.256	0.084	3.045	0.002
Education	0.169	0.102	0.970	0.584	1.662	ns.
Marital Status	−0.094	0.103	−0.744	0.816	−0.912	ns.
Types of Violence/Abuse	0.013	0.107	0.000	0.002	0.125	ns.
Perpetrator	−0.247	0.097	−1.995	0.787	−2.534	0.01
Time	−0.019	0.115	−0.022	0.132	−0.167	ns.

**Table 4:** Kruskal-Wallis ANOVA by Ranks of scores in the ERS

Independent (grouping) Variable: Perpetrator Kruskal-Wallis Test: H(3, *n* = 52) = 11.691; *p* = 0.008				
	Mean	St. Dev.	Ranks Sum	Mean Rank
Husband	34.737	5.197	979.000	25.763
Intimate Partner	38.667	4.899	340.500	37.833
Father	21.000	6.928	13.500	4.500
Mother	35.000	0.010	45.000	22.500

**Table 5:** Multiple comparisons of scores in the ERS considering the perpetrator

Independent (grouping) Variable: Perpetrator Kruskal-Wallis Test: H(3, *n* = 52) = 11.691; *p* = 0.008				
	1. Husband (34.737)	2. Intimate Partner (38.667)	3. Father (21.000)	4. Mother (35.000)
1. Husband (34.737)		0.01	0.005	ns.
2. Intimate Partner (38.667)	0.01		0.00001	ns.
3. Father (21.000)	0.005	0.00001		ns.
4. Mother (35.000)	ns.	ns.	ns.

## Discussion

In the discussion of the results, it will be difficult to refer to results of other research in that field as the authors of this study have not found studies dedicated to that issue in available literature.

The majority of women had higher education; maybe it was their higher education that made them seek help in the CIC. For instance, a complete opposite of such an attitude is the phenomenon consisting in the fact that lower caste women in India are not only more likely to experience discrimination and violence, but they are also more likely to accept unequal treatment and violence as fact of life, keep it to themselves and do not seek help [33, 34]. Besides, seeking help may also be an attempt at saving one’s marriage.

Similar results, i.e. psychological violence as the most commonly experienced type of violence, were also received in other research [11]. Although literature mentions psychological, physical, sexual and other forms of violence, they all actually come down to psychological one: firstly, as soon as any other type of violence takes place, it automatically also becomes psychological one; secondly, consequences of every violence type are psychological too.

The lower ERS scores may indicate that experienced violence weakened or even damaged the women’s resilience, which is of crucial importance for the process of recovery from trauma [11, 28, 35]. That seems logical and consistent, but literature data are not unambiguous. On the one hand, some authors mention adverse consequences of domestic violence in the form of psychological [cf. 36–40] and physical functioning disturbances [cf. 39, 40]; on the other hand, there are studies indicating that women who have ended a battering relationship demonstrate high levels of overall resilience [cf. 11, 41]; and even show both psychological distress and resilience too [cf. 11, 21]. Those studies, however, concern women who were already physically separated from the violence perpetrator (divorced or sheltered), whereas a majority of women experiencing domestic violence in the present study still lived with the perpetrator when the research was carried out.

As we observed, the regression for resilience indicated the age and the person of the perpetrator (with the minus sign) as significant variables. The issue of age seems rather clear: as the woman gains life experience, including by living with the perpetrator, her resilience increases. In turn, among all the studied variables, the weakening or even damaging (the minus sign) of resilience in women experiencing domestic violence is mainly determined by the person of the perpetrator. That is an interesting result worth closer examination.

The above-mentioned ANOVA by ranks and multiple comparisons indicated that violence used by the father most considerably weakened or damaged resilience in the women. The most deleterious impact on their resilience was exerted by violence on the part of the father. That is interesting as the father was not the person most commonly reported as one committing violence against the women (the most common perpetrators were the husband and the intimate partner). Not even referring to Freud’s concept [42] of God being a grand sublimation of father, it is known that the father is a very important person for every human, and thus also violence inflicted by him is more strongly felt and leads to more profound consequences, which may be exemplified and reflected by low resilience. In other words, domestic violence on the part of the father may severely weaken/damage resilience since the father is one of the very close people, very significant others and attachment to him and emotional bonds with him are stronger, and expectations from him are higher, hence the greater disappointment, disenchantment, frustration and psychological suffering caused by violence committed by him. It should be noted that violence used by the mother did not exert such a powerful impact on the study women’s psychological resources.

In turn, the highest resilience occurred in women experiencing violence on the part of the intimate partner (not the husband); it is worth pondering over because both the husband and the intimate partner are individuals with whom the woman remains in an intimate relationship, and thus very close ones. Violence employed by the intimate partner may weaken/damage resilience to a lesser extent than violence on the part of the father and the husband for at least two reasons. Quite possibly, awareness of splitting with the intimate partner being easier (awareness of reversibility of the state of affairs, hence a way out of the difficult situation) than with the husband makes resilience in the women less weakened/damaged by violence. On the other hand, that involves social, cultural and religious issues. Poles (the study concerned Polish women) are mostly Catholics, especially as one of the latest popes was a Pole. An internal (subjective) factor is the interiorisation of certain norms, principles and values such as, for example, the sacrament of marriage. An external (objective) factor is considerable difficulties in obtaining Church-granted divorce or annulment of marriage by the Church. The factors may bring in the women a sense of being condemned to the relationship, of no way out, irrespective of their will, which may breed a feeling of helplessness being very unfavourable for psychological resources [43]. On the other hand, in the case of the intimate partner, with no or weak impact of the above-mentioned internal and external factors, there is a prospect for the possible ending of a battering relationship and hope of freeing oneself from violence, hence possible higher resilience. The processes may not occur in a relationship with the husband, and much less with the father, and thus violence inflicted by them (particularly by the father) so greatly weakens or damages resilience. It seems that definite answers to the above questions may be provided by results of further studies.

It stems from the above that, along with the earlier known adverse consequences of domestic violence experienced by women in the form of disturbed psychological functioning and physical health, resilience is damaged by that too, which ought to be taken into account when devising and implementing help schemes for those women. Therefore, it seems advisable to also consider that aspect of the psychological functioning of women experiencing domestic violence in actions aimed at providing them with psychosocial help.

## Conclusions

Recapitulating the above findings, one can state that, in the study group of women, psychological violence and physical violence were the most commonly experienced forms of violence, while the person most often inflicting violence against them was the husband. Resilience of study women suffering domestic violence was lower than resilience of the general population, not experiencing domestic violence. Experienced violence committed by the father exerted the greatest adverse impact on resilience. The above results may prove useful in therapeutic and prophylactic work with women suffering domestic violence.

### Limitations

The sample size may be a possible limitation.

## Footnotes

1 “Blue Card” is an important part of the Polish system of intervention strategies against domestic violence existing in Poland since 1998 and is filled at an intervention site in the presence of the perpetrator.
